# Dual role of autophagy in gouty arthritis (GA): molecular mechanisms and therapeutic potential

**DOI:** 10.3389/fimmu.2026.1850554

**Published:** 2026-07-15

**Authors:** Marwa Qadri

**Affiliations:** 1 Department of Pharmacology and Toxicology, College of Pharmacy, Jazan University, Jazan, Saudi Arabia; 2 Health Research Center (HRC), Jazan University, Jazan, Saudi Arabia

**Keywords:** autophagic flux, autophagy, gout arthritis, IL-1β, monosodium urate crystals, NLRP3 inflammasome, synovial immune cells, therapeutic targeting

## Abstract

Autophagy is a conserved lysosomal degradation pathway with complex and context-dependent effects in gouty arthritis (GA). Emerging evidence indicates that the autophagic response is determined primarily by the integrity of autophagic flux rather than autophagic activation. Preserved autophagic flux may contribute to inflammatory resolution, whereas impaired autophagic degradation or dysregulated autophagy may promote inflammasome activation, pro-inflammatory cytokine production, and joint tissue damage. These contrasting effects are largely determined by cellular context, as autophagy exhibits varied responses regulated by distinct signaling pathways in neutrophils, monocytes/macrophages, chondrocytes, and osteoblasts. This review summarizes the current evidence on the dual role of autophagy in monosodium urate-induced inflammation, highlights the challenges associated with interpreting autophagic flux, discusses emerging pharmacological and molecular regulators of autophagy in GA, and supports the development of targeted, mechanism-based therapeutic strategies.

## 1 Introduction

Gouty arthritis (GA) is a prevalent inflammatory joint disorder caused by the accumulation of monosodium urate (MSU) crystals in synovial tissues, a consequence of chronic hyperuricemia (1–4). Once deposited, MSU functions as a potent danger signal, initiating robust innate immune responses (2, 5, 6). These responses are characterized by the activation of the NLRP3 inflammasome, the release of pro-inflammatory cytokines such as interleukin-1β (IL-1β), and the rapid recruitment of neutrophils and monocytes to the affected joint (2, 5, 6). Although acute gout flares are generally self-limiting, recurrent episodes and persistent inflammatory signaling contribute to chronic synovitis, cartilage degradation, and joint damage if inadequately managed (7). Current GA management focuses on lifestyle modification and treating acute gout flares with nonsteroidal anti-inflammatory drugs, colchicine, corticosteroids, and urate-lowering agents to prevent recurrent attacks (8). However, these agents are inadequate for preventing recurrent flares or addressing underlying mechanisms in refractory cases (8).

Autophagy, a highly conserved lysosomal degradation process (9, 10), has emerged as a crucial regulator of inflammation and cellular homeostasis in gout. It plays a fundamental role in maintaining intracellular homeostasis by removing misfolded proteins, intracellular pathogens, and danger signals (9, 10). Emerging evidence suggests that autophagy plays a dual and complex role in the pathogenesis of GA. In specific immune cell populations, such as neutrophils, MSU may enhance functional autophagic flux, which in turn promotes neutrophil extracellular trap (NET) formation, accelerates the resolution of acute gout flares, and reduces the recurrence of acute symptoms (11–14). Moreover, autophagy may exert its anti-inflammatory function by negatively regulating NLRP3 inflammasome activation and IL-1β production in neutrophils, monocytes, and macrophages (11–14). Conversely, in macrophages, chondrocytes, and osteoblasts, impaired autophagic flux or excessive autophagy is often associated with lysosomal dysfunction, leading to persistent inflammation, apoptosis, and cartilage damage (15–21). Furthermore, studies have found that autophagic activity is significantly elevated in synovial mononuclear cells from patients with gout, in which it may regulate the release of pro-inflammatory cytokines and metabolic markers (16, 18, 22).

The integrity of autophagic flux, rather than autophagosome formation, is a critical determinant of these opposing effects (23). While autophagosome formation may represent an adaptive response to MSU-induced cellular stress, prolonged exposure to MSU crystals may disrupt lysosomal function, impair autophagic degradation, and shift autophagy from a protective to a pathological process (16, 18). This transition highlights the significance of cell-specific regulation in determining autophagic response in GA.

Despite growing evidence linking autophagy to gout pathogenesis, interpreting the existing literature remains challenging. Many studies rely on static measurements of LC3-II accumulation and p62 expression, which may not reliably distinguish enhanced autophagy initiation from impaired autophagic degradation. Alterations in these markers may indicate either enhanced autophagosome formation or impaired downstream lysosomal degradation. This distinction is particularly important in GA, in which MSU crystals can induce autophagic responses while disrupting lysosomal function. As a result, the functional significance of autophagy activation remains incompletely understood and may explain the contradictory reports describing both the protective and pathological roles of autophagy in GA. Therefore, the aim of this review is to provide an overview of the dual role of autophagy in GA by examining the mechanisms underlying its protective and pathological effects, the challenges associated with interpreting autophagic flux, and the potential of autophagy-based therapeutic strategies.

## 2 Overview of autophagy mechanisms

### 2.1 Autophagy machinery

Autophagy is a highly conserved intracellular degradation pathway that maintains cellular homeostasis in mammalian cells by eliminating misfolded proteins, damaged organelles, and invading pathogens through lysosome-dependent degradation (10). There are three forms of autophagy: macroautophagy, microautophagy, and chaperone-mediated autophagy (24). Macroautophagy is the predominant and the best characterized form in which cytoplasmic cargo is sequestered within double-membrane vesicles known as autophagosomes (24). Autophagy is tightly regulated by cellular signaling pathways that sense nutrient and energy status, particularly the mechanistic target of rapamycin (mTOR) and AMP-activated protein kinase (AMPK) (25). Under basal conditions, mTOR suppresses autophagy, whereas cellular stress and nutrient deprivation activate the AMPK pathway, which inhibits mTOR and stimulates the initiation step (25). The initiation step begins with the activation of the ULK1/2–ATG13–FIP200–ATG101 complex, which initiates autophagosome formation (26, 27). This step is followed by the nucleation stage, in which the class III phosphatidylinositol 3-kinase (PI3K) complex, composed of beclin-1 (ATG6), VPS34, VPS15, and ATG14L, generates phosphatidylinositol-3-phosphate (PI3P) to recruit downstream cytosolic material and promote the formation of the phagophore membrane, a small double-membrane structure isolated from the endoplasmic reticulum (ER) (26). The elongation and maturation of the phagophore are mediated by two ubiquitin-like conjugation systems: the ATG12–ATG5–ATG16L1 complex and the microtubule-associated protein 1 light chain 3 (LC3) conjugation system (28, 29). In the LC3 conjugation system, the cytosolic LC3-I is lipidated by ATG7 and ATG3 to form LC3-II, which promotes its integration into the autophagosomal membrane (28, 29). The autophagosome marker LC3-II acts as a docking site that recruits ubiquitinated cargo via autophagy receptors, such as sequestosome 1/p62 (SQSTM1/p62) for selective degradation (29, 30). Following elongation, the autophagosome matures and fuses with lysosomes to form an autolysosome (24). Acidic hydrolases degrade the engulfed cargo and recycle the resulting components back into the cytoplasm for cellular biosynthesis (24). Through these regulated steps, autophagy preserves cellular integrity, regulates metabolism, and modulates immune and inflammatory responses (24).

### 2.2 Challenges in interpreting autophagic flux in GA

A major challenge in GA research is determining whether increased autophagy-related markers reflect enhanced autophagic activity or impaired downstream degradation. Autophagic flux is a multistage process that includes autophagosome formation, lysosomal fusion, cargo degradation, and recycling, rather than the static accumulation of autophagy-related markers (10, 24). MSU crystals may cause a temporal shift from the initial induction of autophagy to impaired late-stage degradation, resulting in the accumulation of immature autophagosomes (16). In many MSU-induced inflammation studies, the assessment of autophagy relies primarily on static measurements of LC3-II accumulation and p62/SQSTM1 expression (11–22, 31, 32). However, increased LC3-II levels do not necessarily indicate functional autophagic flux, as autophagosome accumulation may result from either enhanced autophagosome formation or defective lysosomal degradation (33–35). Likewise, although p62 accumulation is frequently interpreted as evidence of impaired degradative flux, p62 expression may also be transcriptionally regulated during inflammatory stress (36–38).

This complexity was highlighted by Chen et al., who reported that MSU-exposed macrophages undergo an early increase in autophagosome formation, followed by impaired degradative flux due to lysosomal dysfunction and cathepsin D inactivation (16). Importantly, interpretation of autophagic flux requires integrated multimethod approaches, including lysosomal inhibition assays using bafilomycin A1 or chloroquine, tandem fluorescent LC3 reporter systems, or direct evaluation of lysosomal degradative activity (33, 35, 39, 40). Nevertheless, several studies using GA models rely on measuring LC3-II and p62 as static markers rather than on the dynamic assessment of autophagic flux (11–22, 31, 32). Consequently, methodological limitations in assessing autophagic flux may partly account for the conflicting findings on whether autophagy exerts protective or pathological effects in GA. A dynamic assessment of autophagic flux is therefore essential for accurately defining the role of autophagy in GA (23).

These limitations are particularly relevant in the context of MSU-induced inflammation, in which lysosomal destabilization, oxidative stress, and inflammasome activation may independently alter autophagosome accumulation (16, 17, 20). Functional autophagic flux appears to suppress NLRP3 inflammasome activation by promoting the degradation of damaged mitochondria, reactive oxygen species (ROS), and inflammasome-associated cargo (12, 16, 17, 20). By contrast, defective lysosomal degradation may lead to the accumulation of p62-positive aggregates and amplify inflammatory signaling and cellular injury (16, 17, 20). Therefore, the preservation of autophagic flux integrity, rather than autophagosome formation alone, may represent the critical determinant governing whether autophagy exerts protective or pathological effects in GA.

## 3 Autophagy in gouty arthritis

### 3.1 Protective autophagy in MSU-Induced Inflammation

Neutrophils are among the earliest innate immune cells recruited following MSU crystal deposition and play central roles in both the initiation and spontaneous resolution of acute gouty inflammation (41, 42). Evidence suggests that preserved autophagic flux may contribute to this protective response by regulating NET formation and limiting excessive inflammatory signaling (43). However, the contribution of NETosis to gout pathogenesis appears to be context-dependent, as NETs may initially enhance the inflammatory response by releasing pro-inflammatory mediators, while later contributing to cytokine degradation and the resolution of inflammation (43). Mitroulis et al.’s study was among the first to link autophagy-related signaling to NET formation in GA, showing that neutrophils stimulated with MSU have higher endogenous LC3B expression than unstimulated cells (32). NETs generated following MSU stimulation contain the alarmin high-mobility group box 1 (HMGB1), supporting the pro-inflammatory role of neutrophils in the early phase of gouty attack (32). Importantly, inhibition of PI3K signaling or blockade of phagolysosomal fusion was associated with reduced NET formation, suggesting that autophagy-related machinery may contribute to NETosis in MSU-stimulated neutrophils (32). A limitation of this study is that autophagy was evaluated primarily by LC3B accumulation, whereas downstream degradative activity and lysosomal integrity were not directly assessed. Therefore, whether NET formation reflects preserved autophagic degradation or accumulation of immature autophagosomes remains incompletely understood.

Subsequent studies further expanded the mechanistic relationship between autophagy and NETosis in GA. Huang et al. (12) demonstrated that MSU stimulation enhances neutrophil infiltration, NET formation, and the expression of autophagy-related proteins, including LC3, ATG5, and ATG7, in both murine and human neutrophils (12). Aggregated NETs were associated with reduced levels of IL-1β, TNF-α, MCP-1, and IL-6 in murine air pouch models. These results support the concept that NET-mediated sequestration and degradation of pro-inflammatory mediators may contribute to the spontaneous resolution of acute gout flares (12). Mechanistically, the authors proposed that ATG7 interacts with p53 to facilitate PAD4 transcription and NET formation (12). PAD4 deficiency markedly reduced NETosis and exacerbated the severity of inflammation *in vivo* (12). Collectively, these findings suggest that autophagy-driven NET formation may exert protective effects during acute MSU-induced inflammation. Nevertheless, the underlying evidence is derived mainly from *in vitro* studies and acute experimental models, which may not adequately reflect the inflammatory and metabolic characteristics of chronic gout.

Autophagy also appears to exert protective effects in monocytes and macrophages by inhibiting inflammasome activation and pro-inflammatory cytokine production. Jing Nie and Hongbin Qiu (13) demonstrated that dual-specificity phosphatase 1 (DUSP1), a negative regulator of MAPK signaling, is significantly upregulated in peripheral blood mononuclear cells (PBMCs) from patients with gout and in MSU-stimulated THP-1 monocytes (13). Increased DUSP1 expression was accompanied by elevated LC3/LC3-II levels and reduced p62 accumulation (13). The anti-inflammatory effects of DUSP1 were associated with reduced oxidative stress and lower production of IL-1β, TNF-α, and IL-6 (13). Moreover, pharmacological inhibition of autophagy using 3-methyladenine partially reversed these protective effects, indicating that autophagy-related mechanisms may contribute to DUSP1-mediated suppression of MSU-induced inflammation (13). However, DUSP1 regulates multiple MAPK-dependent inflammatory pathways beyond autophagy (44), making it difficult to determine the specific contribution of autophagy to the observed anti-inflammatory response.

In addition to DUSP1-mediated regulation, Sirtuin 1 (SIRT1)-dependent autophagy has emerged as another potentially protective mechanism in MSU-induced inflammation. SIRT1 is an NAD-dependent deacetylase that modulates cellular metabolism, stress responses, and inflammation through post-translational modification of transcription factors (45). Yang et al. (14) reported that SIRT1 expression is reduced in PBMCs from patients with gout, whereas pharmacological activation of SIRT1 increases LC3 and Beclin-1 expression and suppresses NF-κB translocation and IL-1β production in MSU-stimulated PBMCs (14). Similarly, Hsieh et al. (11) demonstrated that a synthetic SIRT1 activator attenuates NLRP3 inflammasome activation, caspase-1 cleavage, and IL-1β release in human and murine macrophages while simultaneously reducing lysosomal rupture and mitochondrial damage (11). These findings suggest that the preservation of cellular homeostasis may mitigate MSU-induced inflammation by maintaining lysosomal integrity, suppressing oxidative stress, and inhibiting inflammasome activation. Importantly, the pleiotropic nature of SIRT1 signaling (46–50) suggests that the reported protective effects are likely mediated by the regulation of multiple cellular pathways rather than by autophagic modulation alone.

Collectively, current evidence suggests that the preservation of autophagic homeostasis in neutrophils and monocyte/macrophage populations may contribute to the resolution of acute MSU-induced inflammation by regulating NET formation, lysosomal stability, oxidative stress, and inflammasome signaling. However, the protective effects of autophagy are likely to vary according to cell type, inflammatory state, and the integrity of degradative flux.

### 3.2 Pathological Autophagy in MSU-Induced Inflammation

While autophagy functions as a protective mechanism that limits the inflammatory response in GA, accumulating evidence suggests that excessive autophagy or impaired autophagic flux may contribute to joint tissue damage and the amplification of pro-inflammatory signaling pathways. In particular, MSU-induced autophagy has been implicated in the pathological responses of synovial immune cells, chondrocytes, and osteoblasts, highlighting the context-dependent nature of autophagy in GA.

Evidence supporting the detrimental role of autophagy has been demonstrated in human chondrocytes (19). MSU crystals may contribute significantly to cartilage damage via the enhancement of pro-inflammatory mediators and cartilage-degrading enzyme production (19). Hwang et al. (19) demonstrated that exposure of human articular chondrocytes isolated from patients with OA to MSU reduces chondrocyte survival in a time- and dose-dependent manner (19). MSU stimulation was associated with increased LC3-II accumulation and enhanced autophagosome formation, together with suppression of the Akt-mTOR pathway, a major negative regulator of autophagy (19). Moreover, pharmacological inhibition of autophagosome formation partially restored chondrocyte survival (19). Interestingly, the reduction in cell viability was not accompanied by significant activation of apoptosis or ER stress-associated markers, indicating that autophagy-related signaling may represent a distinct mechanism of cellular injury in this setting (19). These findings suggest that excessive autophagic activity may contribute to chondrocyte death and cartilage degeneration during GA (19). However, these findings were derived from short-term chondrocyte cultures and, therefore, may not fully capture the persistent inflammatory alterations observed in GA.

Similarly, Xiao et al. (21) identified SRY-box transcription factor 8 (SOX8), a cell-fate regulator, as an important determinant of chondrocyte survival (21). Using MSU-treated human C28/I2 chondrocytes and an acute rat gout model, the authors demonstrated that MSU exposure suppresses SOX8 expression, increases the LC3-II/I ratio, and promotes chondrocyte apoptosis (21). Restoration of SOX8 expression partially reversed these effects by activating the PI3K-Akt-mTOR pathway and reducing autophagy-associated markers (21). However, the extent to which LC3-II/I alterations reflected functional autophagy remains uncertain, as degradative flux and lysosomal activity were not comprehensively assessed.

Pathological autophagy has also been implicated in osteoblast dysfunction during chronic GA (15). Allaeys et al. (15) demonstrated that exposure of human osteoblasts to MSU crystals promotes crystal internalization and induces activation of NLRP3-associated autophagy (15). These changes were accompanied by increased IL-1β production and matrix metalloproteinase activity, impaired osteoblast proliferation, and reduced bone-forming capacity (15). The findings suggest that autophagy-associated inflammatory signaling may contribute to impaired bone remodeling and joint damage in chronic gout. However, the osteoblast response to MSU crystals likely involves the activation of multiple inflammatory signaling pathways beyond autophagic alteration alone, including ROS generation and inflammasome activation, highlighting the cell type-dependent effects of autophagy in GA (15).

The pathological role of autophagy is not limited to chondrocytes and osteoblasts. Defects in autophagic degradation or flux have also been reported in human monocytes/macrophages exposed to MSU crystals (16). Chen et al. (2023) investigated autophagy dynamics in synovial monocytes/macrophages from gouty patients and peripheral monocytes from healthy donors, as well as *in vitro* RAW264.7 macrophages stimulated with MSU crystals (16). The study demonstrated that stimulation of these cells with MSU initially triggered autophagosome formation but subsequently led to impaired autophagic flux due to lysosomal dysfunction (16). Interestingly, the study found that synovial fluid monocytes from patients with acute gouty attacks showed elevated levels of both LC3B-II and p62, indicating the accumulation of autophagosomes that do not undergo lysosomal degradation, whereas only elevated p62 levels were present in synovial tissues from patients with chronic GA (16). Furthermore, human monocytes/macrophages and murine macrophages exhibited a biphasic autophagic response following MSU exposure (16). During the early phase, MSU induced autophagosome formation, as supported by increased LC3-II expression and the accumulation of autophagic vesicles, indicating activation of the autophagy machinery in response to cellular stress (16). However, during the late phase, lysosomal dysfunction impaired autophagic flux, resulting in defective cargo degradation, as reflected by increased LC3-II and p62 expression (16). Specifically, MSU crystals inactivated the lysosomal protease cathepsin D, a key enzyme required for autolysosomal degradation (16). Consequently, the autophagic degradation marker p62 accumulated, indicating defective autophagic clearance (16). This disruption of autophagic degradation was associated with macrophage apoptosis (16). Cumulatively, these results indicate that, although MSU crystals may initially trigger autophagy as an adaptive response, persistent exposure may lead to defective autophagic flux in monocytes/macrophages at later stages, thus contributing to macrophage apoptosis and exacerbating synovial inflammation in GA (16). Compared with earlier studies, this work provided a more comprehensive evaluation of degradative dysfunction by assessing lysosomal activity and cargo accumulation; however, additional dynamic flux assays would further strengthen the mechanistic interpretation of the temporal transition from adaptive autophagy to defective degradation.

Consistent with this mechanism, subsequent studies have identified p62 accumulation as an important regulator linking defective autophagy to NLRP3 inflammasome activation in GA (17, 20). Kim/Choe studies (2013/2014) demonstrated that MSU stimulation promotes p62 accumulation in human monocytes and RAW264.7 macrophages, along with increased caspase-1 activation and IL-1β production (17, 20). These studies proposed that accumulated p62 activates ERK and JNK signaling pathways and amplifies NLRP3 inflammasome-mediated inflammatory responses (17, 20). These observations support the idea that defective autophagic degradation may contribute to persistent inflammasome activation in GA (17, 20). However, p62 is also involved in multiple inflammatory signaling pathways independent of autophagy (36–38), making it difficult to determine whether these effects are solely related to impaired autophagic flux.

In addition to the MSU crystal, soluble uric acid itself may skew monocytes to a pro-inflammatory phenotype by suppressing autophagy (31). Crişan et al. (31) demonstrated that priming human monocytes isolated from healthy individuals with soluble uric acid increased IL-1β production and reduced IL-1 receptor antagonist (IL1Ra) levels (31). Transcriptomic analysis revealed activation of the AKT-PRAS40-mTOR pathway, consistent with suppression of autophagy-related signaling and reduced LC3-II expression (31, 51). Raptor dissociates from mTOR complex 1, thereby activating mTOR signaling, which in turn inhibits autophagy (51). These findings expand the pathogenesis of GA beyond acute crystal-induced inflammation and suggest that chronic hyperuricemia may establish a chronic pro-inflammatory state before crystal deposition occurs. However, the relationship between soluble uric acid-mediated metabolic reprogramming and functional autophagic flux remains insufficiently characterized in chronic GA.

Metabolic disturbances within the gouty microenvironment may further contribute to autophagy dysregulation (18). Fu et al. (2023) used integrated proteomic and metabolomic analyses to identify the accumulation of lipid metabolites, increased phospholipase A2 (PLA2) expression, and impaired lysosomal acidification in synovial fluid from patients with gouty knee arthritis (18). These changes were associated with altered LC3B and p62 expression profiles, supporting the disruption of autophagic degradation pathways (18). Similarly, Guo et al. (22) demonstrated that circ_0058051 expression is increased in PBMCs from patients with recurrent gout, whereas miR-129-5p expression is reduced and ATG7 expression is elevated (22). Overexpression of ATG7 in MSU-stimulated THP-1 macrophages was associated with increased production of IL-1β, TNF-α, and IL-6, suggesting a possible link between dysregulated noncoding RNA signaling and autophagy-related inflammatory responses (22). However, the extent to which these effects are mediated directly through autophagy-related mechanisms remains incompletely defined.

Taken together, these studies indicate that pathological autophagy in GA is highly heterogeneous, involving either excessive autophagy in chondrocytes and osteoblasts or impaired degradative flux in inflammatory monocytes and macrophages. Interpretation of the current literature remains limited by methodological variability, reliance on static LC3-II and p62 measurements, and insufficient assessment of dynamic autophagic flux. Furthermore, most available evidence is derived from acute *in vitro* systems or acute murine MSU models, which may not fully recapitulate the chronic inflammatory and metabolic context of human GA. Future studies employing rigorous flux analyses, lysosomal functional assays, and clinically relevant chronic disease models are therefore needed to clarify the precise contribution of autophagy dysregulation to GA progression. The key studies supporting the dual role of autophagy in GA are presented in **Table 1**.

**Table 1:** Experimental evidence supporting the protective and pathological roles of autophagy in GA and key methodological considerations.

Study	Experimental model	Main findings	Major limitation	Interpretation
Mitroulis et al., 2011 (32)	MSU-stimulated human neutrophils	MSU stimulation increased LC3B expression and promoted NET formation; inhibition of autophagy-related signaling reduced NET release	LC3B expression used as surrogate marker; dynamic flux not directly assessed	Autophagy-related signaling appears to contribute to NET formation during acute gout inflammation
Huang et al., 2023 (12)	MSU-stimulated human neutrophils and murine air pouch model	ATG7-mediated autophagy enhanced NET formation and reduced IL-1β, TNF-α, MCP-1, and IL-6 in murine gout models	Focused on acute inflammation model; chronic disease relevance remains unclear	Preserved neutrophil autophagy may facilitate inflammatory resolution during acute gout
Nie and Qiu, 2024 (13)	MSU-stimulated human monocytes	DUSP1 activation enhanced autophagy, reduced mitochondrial injury, and suppressed NLRP3 inflammasome activation	Primarily mechanistic *in vitro* sitting, and DUSP1 exerts broad anti-inflammatory effects through multiple signaling pathways, making the specific contribution of autophagy difficult to isolate	Autophagy may limit inflammation through NLRP3 suppression
Yang et al., 2019 (14)	SIRT1 activation in MSU-stimulated human monocytes	Resveratrol increased SIRT1 expression, enhanced autophagy-related signaling, and reduced IL-1β production in gout PBMCs	Primarily mechanistic *in vitro* evidence and multiple anti-inflammatory pathways involved beyond autophagy	SIRT1-associated autophagy enhancement may contribute to suppression of gout inflammation
Chih-Yu Hsieh et al., 2020 (11)	SIRT1 activation in MSU-stimulated human and murine monocytes/macrophages	4-HAB induced SIRT1-dependent autophagy, reduced mitochondrial damage and lysosomal rupture, and inhibited NLRP3 activation	Pleiotropic anti-inflammatory effects of 4-HAB	Enhanced autophagy is associated with attenuation of inflammasome-mediated inflammation
Hwang et al., 2015 (19)	MSU-stimulated human osteoarthritic chondrocytes	MSU crystals increased LC3-II accumulation, inhibited AKT/mTOR signaling, and induced autophagy-associated chondrocyte death	Short-term *in vitro* model; flux not comprehensively assessed	Excessive autophagy-related activity may contribute to chondrocyte injury following MSU exposure
Xiao et al., 2023 (21)	MSU-stimulated human C28/I2 chondrocytes; acute gout rat model *in vivo*	MSU crystals suppressed SOX8 expression, inhibited PI3K/AKT/mTOR signaling, increased autophagic activity, and promoted cartilage damage	Reliance on LC3-II measurements without flux assessment	Dysregulated autophagy may contribute to cartilage degeneration in gout
Allaeys et al., 2013 (15)	MSU-stimulated human osteoblasts	MSU crystals induced NLRP3-dependent autophagy in osteoblasts, reduced mineralization capacity, increased MMP activity, and impaired osteoblast function	Simultaneously activated multiple osteoblast signaling pathways, making the specific contribution of autophagy difficult to determine	Autophagy-related responses in osteoblasts may contribute to defective bone remodeling in chronic gout
Chen et al., 2023 (16)	MSU-stimulated gout monocytes and RAW264.7 macrophages	MSU crystals induced early autophagosome formation followed by lysosomal dysfunction, cathepsin D inactivation, p62 accumulation, impaired autophagic degradation, and macrophage apoptosis	Further dynamic flux studies needed to define temporal progression	There is a strong evidence that impaired degradative flux may promote persistent inflammation and cellular injury
Choe et al., 2014 and Kim et al., 2013 (17, 20)	MSU-stimulated THP-1 monocytes and RAW 264.7 macrophages	MSU crystals promoted p62 accumulation, ERK/JNK activation, caspase-1 activation, and enhanced IL-1β production	Autophagy-independent signaling functions of p62, complicating the interpretation of its accumulation as a marker of impaired autophagy	p62 accumulation is linked with the amplification of inflammatory signaling during MSU-induced inflammation
Crişan et al., 2017 (31)	Soluble uric acid- stimulated human monocytes	Soluble uric acid activated AKT-PRAS40-mTOR signaling, suppressed autophagic activity, reduced IL-1Ra production, and enhanced inflammatory priming	Did not directly evaluate autophagic flux; focused on soluble uric acid rather than crystal-induced gout	Hyperuricemia may promote inflammatory priming through suppression of autophagy before crystal deposition
Fu et al., 2023 (18)	Human gout synovial fluid (proteomics/metabolomics)	Proteomic and metabolomic analyses identified lipid accumulation, lysosomal dysfunction, altered autophagy markers, and disrupted autophagy-lysosome homeostasis in gout synovial fluid	Associative rather than causal evidence; requires further mechanistic clarification	Findings may support a metabolic contribution to autophagy dysregulation in gout
Guo et al., 2025 (22)	Gout PBMCs; MSU-stimulated gout PBMCs and THP-1 monocytes	circ_0058051 upregulated ATG7 expression and increased LC3, IL-1β, TNF-α, and IL-6 levels in recurrent gout models.	Focused primarily on ATG7 and LC3 expression without comprehensive assessment of autophagic flux	Non-coding RNA-mediated regulation of autophagy-related pathways may contribute to recurrent gout inflammation

### 3.3 Cell-specific and stage-specific roles of autophagy in GA

Autophagy in GA is a dynamic, context-dependent process. Its exact role depends on the specific cell type involved and disease stage, determining whether autophagy promotes survival or drives inflammation. Preserved autophagic flux in neutrophils and monocytes/macrophages serves as a vital cytoprotective mechanism. It maintains innate immune homeostasis by selectively clearing damaged organelles, preventing oxidative stress, dampening excessive pro-inflammatory cascades, and facilitating the safe degradation of inflammatory debris (11–14, 16). Specifically, in neutrophils, autophagy is a crucial molecular regulator of NET formation and cytokine clearance in MSU-induced sterile inflammation (12). These mechanisms may contribute to the resolution of inflammation and the restoration of tissue homeostasis.

By contrast, chondrocytes and osteoblasts are specialized, long-lived structural cells with limited regenerative capacity that rely on precise intracellular metabolic homeostasis (52, 53). Under persistent MSU exposure or chronic inflammatory stress, excessive autophagic activity or impaired lysosomal degradation may promote cellular dysfunction, apoptosis, and defective extracellular matrix maintenance and bone remodeling (15, 19, 21). Furthermore, accumulation of LC3-II- and p62-positive autophagosomes in structural and inflammatory cells has been associated with the amplification of inflammatory signaling and tissue injury (15, 19, 21). These observations suggest that autophagy may become detrimental when degradative capacity is chronically impaired by sustained cellular stress.

The disease stage may further alter autophagic responses in GA. During acute gout flares, autophagy may predominantly function as a transient adaptive mechanism that facilitates inflammasome degradation and restoration of cellular homeostasis (11–14, 16). However, recurrent MSU exposure, chronic hyperuricemia, and persistent lysosomal dysfunction may progressively impair autophagic flux and contribute to chronic inflammation and impaired tissue remodeling (15, 18, 19, 21, 31). Furthermore, systemic hyperuricemia may induce immune priming in circulating monocytes, even in the absence of crystal deposition, and alter basal autophagy-related signaling before the onset of acute gout attacks (31). Therefore, autophagy-mediated responses in GA are governed by crosstalk between flux integrity and the cellular environment.

### 3.4 Autophagy crosstalk in gouty arthritis

The dual role of autophagy results from complex crosstalk among inflammatory signaling pathways, metabolic regulators, and cell-specific factors that determine whether autophagic activity is protective or pathogenic (12–22, 31, 32). MSU crystals represent a common stimulus that initially activates autophagy across multiple cell types in the joint environment, including chondrocytes, joint-resident macrophages, monocytes, neutrophils, and osteoblasts (12–22, 31, 32), but ultimately elicit different responses depending on the integrity of autophagic flux. Autophagic flux refers to the degradation of cellular cargo and the maintenance of cellular homeostasis (24). When autophagic flux remains functional, autophagy contributes to the resolution of inflammation and the clearance of MSU crystals (12–14). By contrast, impaired autophagic degradation leads to the accumulation of autophagy-related proteins and amplifies the inflammatory response to MSU crystals (15–22, 31).

A key feature of this regulatory network is the interaction between autophagy and the NLRP3 inflammasome. The NLRP3 inflammasome modulates inflammatory responses and interacts with multiple autophagic pathways (54, 55). In neutrophils and monocytes/macrophages, preserved autophagic flux may negatively regulate NLRP3 inflammasome activation by removing intracellular stress signals and degrading inflammasome-associated components (12–14). In MSU-stimulated neutrophils, autophagy has also been implicated in regulating NET formation and cytokine degradation during acute gout flares via the ATG7-p53-PAD4 signaling axis (12). Similarly, activation of the DUSP1 and SIRT1 signaling pathways has been associated with enhanced autophagic activity and suppression of NLRP3 inflammasome activation in monocytes/macrophages following MSU exposure (13, 14). The preserved degradative flux may function as an adaptive mechanism that restrains excessive innate immune activation during early inflammatory responses. Conversely, defective lysosomal function in monocytes/macrophages may promote NLRP3 and caspase-1 activation, leading to sustained IL-1β production via activation of ERK and JNK signaling (17, 20). Lysosomal dysfunction thus emerges as a key driver of defective autophagic flux (16), and metabolic changes within the joint microenvironment, including PLA2 activity, may interfere with lysosomal integrity and disrupt autophagy-lysosome homeostasis (18). Moreover, prolonged exposure to MSU crystals has been associated with lysosomal destabilization, cathepsin D inactivation, p62 accumulation, and impaired autophagic degradation in monocytes/macrophages (16). These alterations are accompanied by sustained activation of ERK/JNK signaling, caspase-1 cleavage, and IL-1β production (17, 20).

Metabolic disturbances within the gouty microenvironment may further aggravate this process. Proteomic and metabolomic studies have linked PLA2-associated lipid dysregulation with impaired lysosomal acidification and disruption of autophagy-lysosome homeostasis in GA (18). In addition, soluble uric acid may exacerbate lysosomal dysfunction by chronically suppressing autophagy through the AKT-PRAS40-mTOR pathway (31). Emerging evidence also suggests that noncoding RNA regulatory networks, including the circ_0058051/miR-129-5p/ATG7 axis, may alter inflammasome-associated inflammatory responses in recurrent gout (22). Collectively, these findings indicate that chronic lysosomal stress and impaired degradative flux may represent important contributors to sustained inflammation and macrophage dysfunction in GA.

Autophagy-related crosstalk may also exert distinct effects on structural joint cells. In chondrocytes, MSU exposure has been associated with the suppression of SOX8 expression and the disruption of PI3K/AKT/mTOR signaling, resulting in excessive autophagic activity, apoptosis, and impaired extracellular matrix maintenance (21). Similarly, NLRP3-associated autophagic responses in osteoblasts have been linked to abnormal inflammatory signaling and defective bone remodeling during chronic gout inflammation (15). In structural joint cells, autophagy may contribute to tissue injury when persistent cellular stress exceeds the degradative capacity of lysosomes.

Overall, autophagy in GA exhibits distinct activity patterns that change throughout disease progression (12–22, 31, 32). Experimental studies indicate that MSU exposure may initially induce adaptive autophagosome formation during early inflammatory responses, whereas persistent crystal exposure may progressively impair lysosomal degradation and disrupt autophagic flux (16). This transition from early adaptive autophagy to late-stage impairment of autophagic flux may contribute to the shift from acute inflammation to chronic tissue damage (16). Targeting these regulatory networks at distinct disease stages, such as DUSP- and SIRT-mediated autophagy activation, PLA2-driven lysosomal dysfunction, or SOX8-mediated transcriptional regulation, may offer effective therapeutic opportunities to limit acute and refractory gout flares beyond IL-1 blockade.

Emerging evidence indicates that autophagy may also interact with extracellular vesicle (EV) biogenesis and lipid raft signaling, providing an additional regulatory mechanism beyond conventional intracellular degradation. Recent studies have demonstrated bidirectional crosstalk between autophagy and EV secretion, whereby autophagic activity may regulate EV biogenesis, cargo composition, and release, while EVs can modulate autophagy-related signaling pathways in recipient cells (56, 57). Mechanistically, multivesicular bodies (MVBs) serve as critical intersections between autophagic and secretory pathways. Autophagosomes can fuse with MVBs to form amphisomes that subsequently undergo lysosomal degradation; however, when autophagic flux is impaired, undegraded cargo may instead be redirected toward EV secretion (56, 58). Furthermore, lipid rafts, which are cholesterol-rich membrane microdomains involved in signal transduction and vesicular trafficking, have been implicated in regulating autophagy and EV formation (59). Autophagy has been shown to promote the enrichment of lipid raft-associated components within EVs (59), suggesting the coordinated regulation of membrane dynamics and intercellular communication. Although the precise mechanisms remain incompletely understood in GA, this autophagy-EV-lipid raft axis may modulate inflammasome activation and cytokine production. Future studies exploring the interplay between autophagy, EVs, and lipid raft-associated signaling could provide valuable insight into the multifaceted role of autophagy in GA pathogenesis.

### 3.5 Autophagy modulators in GA

Pharmacological targeting of autophagy has emerged as a potential therapeutic strategy for modulating MSU-derived inflammation in GA. A wide range of natural compounds, synthetic agents, and traditional herbal formulations have been shown to modulate autophagic activity across experimental models by restoring autophagic flux, enhancing lysosomal function, reducing excessive autophagy, and suppressing intracellular stress and downstream inflammatory cascades.

Despite their diversity, these agents share common functional responses, including improved lysosomal degradation, reduced inflammasome activation, and pro-inflammatory cytokine secretion, beyond the regulation of autophagy alone, as outlined in **Table 2**.

**Table 2:** Pharmacological modulators of autophagy-related pathways in GA: experimental evidence, proposed mechanisms, and key considerations.

Study/compound	Experimental model	Evidence for autophagy regulation	Anti-inflammatory effects	Proposed mechanism/key consideration
Liu et al., 2025 (Daphnetin) (60)	MSU-stimulated THP-1 macrophages; murine gout model	↑ LC3-II, ↑ Beclin-1, ↓ p62	↓ NLRP3, ↓ IL-1β	AMPK-mTOR-associated autophagic responses were associated with suppression of inflammasome signaling; direct flux assessment was not reported
Liu et al., 2025 (Nobiletin) (61)	MSU-stimulated THP-1 macrophages; murine gout model	↑ LC3-II/LC3-I, ↑ Beclin-1, ↓ p62	↓ NF-κB, ↓ NLRP3, ↓ caspase-1, ↓ IL-1β	Anti-inflammatory effects were associated with AMPK-mTOR activation; the contribution of autophagy-independent pathways cannot be excluded
Lou et al., 2022 (Ursolic acid derivative) (62)	MSU-stimulated THP-1 monocytes	↑ LC3-II/LC3-I, ↓ p62	↓ ROS, ↓ caspase-1, ↓ NLRP3, ↓ IL-1β	Effects may involve both autophagy-related regulation and antioxidant activity
Liu et al., 2023 (Qingre Huazhuo Jiangsuan Decoction) (64)	Rat model of gout	↑ LC3-II, ↓ p62	↓ NLRP3, ↓ IL-1β, improved synovitis	Anti-inflammatory effects were associated with PI3K/AKT/mTOR-related autophagy modulation; evidence remains based on an acute rat model
Han et al., 2021 (Zisheng Shenqi Decoction) (63)	Rat model of gout	↑ LC3-II, ↓ p62	↓ NLRP3, ↓ IL-1β, improved synovitis	Herbal formulation was associated with enhanced autophagy-related responses through PI3K/AKT/mTOR modulation; compound-specific active mechanisms remain unclear
Du et al., 2024 (Triptolide) (65)	RAW264.7 macrophages; neutrophils; murine air pouch model	↓ Beclin-1, ↓ LC3B in neutrophils	↓ NETs and ↑ apoptosis shift macrophage polarization into anti-inflammatory phenotypes.	Suggests that suppression of excessive autophagy-related activity through the PI3K/AKT/mTOR pathway may be beneficial in neutrophils, highlighting the context-dependent effects of autophagy modulation
Yang et al., 2019 (Resveratrol) (14)	MSU-stimulated gout PBMCs	LC3-II unchanged, ↓ Beclin-1	↓ NF-κB, ↓ NLRP3, ↓ IL-1β	Anti-inflammatory activity may primarily reflect SIRT1-mediated signaling rather than direct enhancement of autophagy
Hsieh et al., 2020 (4-HAB) (11)	MSU-stimulated THP-1, J774A.1, BMDM macrophages	↑ LC3-II	↓ IL-1β, ↓IL-18, ↓ caspase-1 and ASC ↓ lysosomal rupture, ↓ mitochondrial damage	Preservation of mitochondrial and lysosomal integrity may contribute to inflammasome suppression
Yuan et al., 2023 (PP121) (66)	MSU-stimulated THP-1macrophages; murine model of gout	↑ LC3-II, ↑ Beclin-1, ↓ p62	↓ pyroptosis, ↓ caspase-1, ↓ IL-1β	Findings may support autophagy-associated degradation of NLRP3 inflammasome components
Piao et al., 2022 (Taxifolin) (67)	MSU-stimulated peritoneal murine macrophages; murine model of gout	↑ LC3-II, ↑ Beclin-1, ↓ p62	↓ caspase-1 ↓ HMGB1, ↓ NLRP3, ↓ IL-1β	Protective effects may involve both enhanced autophagic activity and reduced oxidative stress
Xu et al., 2022 (Tanshinone IIA) (68)	MSU-stimulated neutrophils isolated from the air pouch of C57BL/6 mice; murine model of gout	↓ LC3-II, ↓ Beclin-1	↓ NET formation, ↓ caspase and NLRP3 in ankle joints	Suggests that inhibition of excessive autophagy-related responses may attenuate neutrophil-driven inflammation

↑ indicates increased expression or production, activation, or activity; ↓ indicates decreased expression or production, activation, or activity.

#### 3.5.1 AMPK-mTOR pathway modulators

The AMPK-mTOR signaling axis represents one of the most extensively investigated pathways for pharmacological targeting of autophagy in GA. Activation of AMPK suppresses mTOR signaling and promotes autophagy, which may contribute to attenuating MSU-induced inflammation (54, 55). Several natural compounds have been demonstrated to exert anti-inflammatory effects through this pathway. For instance, the coumarin derivative daphnetin was shown to enhance AMPK activation and suppress mTOR signaling in human macrophages *in vitro* and murine gout models, resulting in reduced inflammasome activation and cytokine production (60). Similarly, the flavonoid nobiletin has been reported to exert comparable effects by promoting AMPK-mTOR-associated autophagic responses together with inhibition of NF-κB signaling in MSU-stimulated human macrophages (61). Triterpenoid derivatives, including ursolic acid-related compounds, have also been associated with reduced oxidative stress and inflammatory cytokine release, accompanied by alterations in AMPK-related signaling pathways (62). In parallel, traditional herbal formulations, such as Qingre Huazhuo Jiangsuan Decoction and Zisheng Shenqi Decoction, have been reported to reduce inflammatory responses in rat models of GA and increase the expression of autophagy-related proteins associated with AMPK-dependent signaling pathways (63, 64). Notably, some compounds appear to exert context-dependent regulatory effects rather than autophagy activation. Triptolide has been reported to suppress excessive autophagy-related activity and NET formation while modulating macrophage polarization through AKT-mTOR-associated signaling (65). However, these studies primarily assess autophagy by measuring LC3-II and p62 expression, without directly evaluating dynamic autophagic flux or lysosomal degradative function. In addition, the broad anti-inflammatory actions of these compounds indicate that their therapeutic effects likely involve multiple signaling pathways beyond autophagy regulation.

#### 3.5.2 SIRT1-dependent modulators

Sirtuin 1 (SIRT1) has emerged as another important regulator linking autophagy, cellular metabolism, and inflammatory signaling (46, 48). Activation of SIRT1 has been linked to the deacetylation of autophagy-related proteins and interactions with AMPK-associated signaling pathways (46, 48). Resveratrol, a natural SIRT1 agonist, has been shown to increase SIRT1 expression in peripheral monocytes from patients with gout, with concomitant enhancement of autophagy-related signaling and reduction of inflammatory cytokine production following MSU stimulation (14). Similarly, 4-hydroxy auxarconjugatin B (4-HAB) has been reported to promote SIRT1-associated autophagic responses in human and murine macrophages *in vitro* and is associated with reduced mitochondrial dysfunction, lysosomal damage, and inflammasome activation (11). These findings suggest that SIRT1-mediated restoration of cellular homeostasis may attenuate MSU-induced inflammatory signaling. However, SIRT1 activators exert broad metabolic and anti-inflammatory effects (46–50). Consequently, the relative contribution of autophagic regulation to the observed anti-inflammatory responses remains incompletely defined. Although SIRT1-targeted therapies have shown promising effects in acute experimental models, their long-term therapeutic effects in the chronic gout model have yet to be determined.

#### 3.5.3 Other autophagy-inflammasome modulators

Several additional compounds have been reported to modulate the interaction between autophagy and inflammasome activation in GA. PP121, a pyroptosis-related compound, has been shown to promote autophagy-associated degradation of NLRP3 inflammasome components in MSU-challenged macrophages and murine gout models, accompanied by attenuation of pyroptotic signaling and IL-1β release (66). Likewise, the natural flavonoid Taxifolin has been linked to enhanced macrophage phagocytic activity, alterations in autophagy-related pathways, and reduced inflammasome activation in murine models of GA (67). In a similar context, Tanshinone IIA, a diterpenoid naphthoquinone found in traditional Chinese medicine, has been associated with reduced autophagy-related activity and NET formation in neutrophils isolated from murine air pouch models, together with attenuation of NLRP3 inflammasome-associated inflammatory responses (68). Overall, the therapeutic effects of these compounds may be influenced by cell type, disease stage, and the integrity of autophagic flux. In addition to autophagy-related pathways, these compounds may regulate oxidative stress, mitochondrial homeostasis, and inflammatory signaling networks that contribute to their overall biological effects.

#### 3.5.4 Emerging molecular and genetic regulators

Emerging evidence suggests that autophagy in GA is additionally regulated through transcriptional and posttranscriptional mechanisms in a cell-specific manner. DUSP1 and SIRT1 have been linked to autophagy-related signaling and reduced inflammasome activity in monocytes/macrophages (11, 13, 14). By contrast, reduced SOX8 expression in chondrocytes following MSU exposure is accompanied by increased autophagic activity and apoptosis, together with alterations in PI3K-AKT-mTOR signaling (21). Additionally, noncoding RNA networks have been implicated in autophagy-associated responses in recurrent gout (22). The circ_0058051/miR-129-5p/ATG7 regulatory axis has been associated with elevated ATG7 expression and increased IL-1β production in MSU-stimulated monocytes/macrophages (22). Although these findings highlight the complexity of autophagy regulation in GA, most of the current literature remains observational. Therefore, future studies incorporating robust analyses of autophagic flux, longitudinal experimental models, and validation in human tissues are needed to establish the translational potential of these molecular targets.

#### 3.5.5 Translational challenges and therapeutic limitations

Translating the pharmacological modulation of autophagy from preclinical models to clinical gout management remains challenging. Existing evidence is derived predominantly from *in vitro* studies and acute MSU-induced animal models. Human GA is characterized by heterogeneous disease presentation, prolonged hyperuricemia, recurrent inflammatory episodes, and metabolic comorbidities, all of which may substantially influence autophagic responses and therapeutic outcomes (1–8).

Another important limitation is the pleiotropic nature of the many compounds discussed in this review. Several natural products and synthetic agents may exert broad anti-inflammatory, antioxidant, and metabolic effects, in addition to modulating autophagy-related pathways. Consequently, distinguishing direct autophagy-dependent mechanisms from parallel effects on ROS generation, inflammasome signaling, or cellular metabolism remains challenging in many experimental studies. Therefore, the observed therapeutic effects are likely mediated through a combination of autophagy-dependent and autophagy-independent mechanisms.

Given the essential role of autophagy in maintaining cellular homeostasis (9, 10), systemic modulation of autophagy may lead to unintended biological consequences across cell populations. While enhancing autophagy may exert anti-inflammatory effects in immune cells, it may simultaneously contribute to apoptosis and tissue damage in structural joint cells under certain conditions. Therefore, future therapeutic strategies should prioritize the selective modulation of autophagy based on cellular context and disease stage.

Future translational studies should focus on the dynamic assessment of autophagic flux in clinically relevant settings, together with the identification of selective modulators that preserve lysosomal integrity without disrupting physiological autophagic homeostasis. A better understanding of disease heterogeneity and cell-specific autophagic responses may facilitate the development of more precise therapeutic strategies targeting autophagy in gout.

## 4 Conclusions and future perspectives

GA is an autoinflammatory arthropathy that sits at the intersection of metabolic dysregulation and innate immune activation, with disease processes initiated by MSU crystal deposition in the joints and amplified through NLRP3 inflammasome signaling. Autophagy has emerged as a pivotal regulator of GA pathogenesis, exhibiting a dual and context-dependent role. Functional autophagic flux may facilitate the resolution of inflammation, particularly in neutrophils and monocytes. By contrast, impaired flux in macrophages, osteoblasts, and chondrocytes may lead to lysosomal dysfunction, sustained NLRP3 activation, pro-inflammatory cytokine production, and progressive joint damage. These observations highlight the importance of distinguishing autophagy activation from functional autophagic flux when interpreting the role of autophagy in gout.

Importantly, many published studies provide only indirect evidence of autophagic activity, thus limiting the accurate evaluation of autophagic degradative function. This methodological limitation may contribute to conflicting findings regarding the protective and pathological effects of autophagy reported across experimental models. However, the available evidence indicates that preservation of autophagic flux integrity, rather than autophagy activation alone, represents a key determinant of autophagy-associated outcomes in GA.

**Figure 1:**
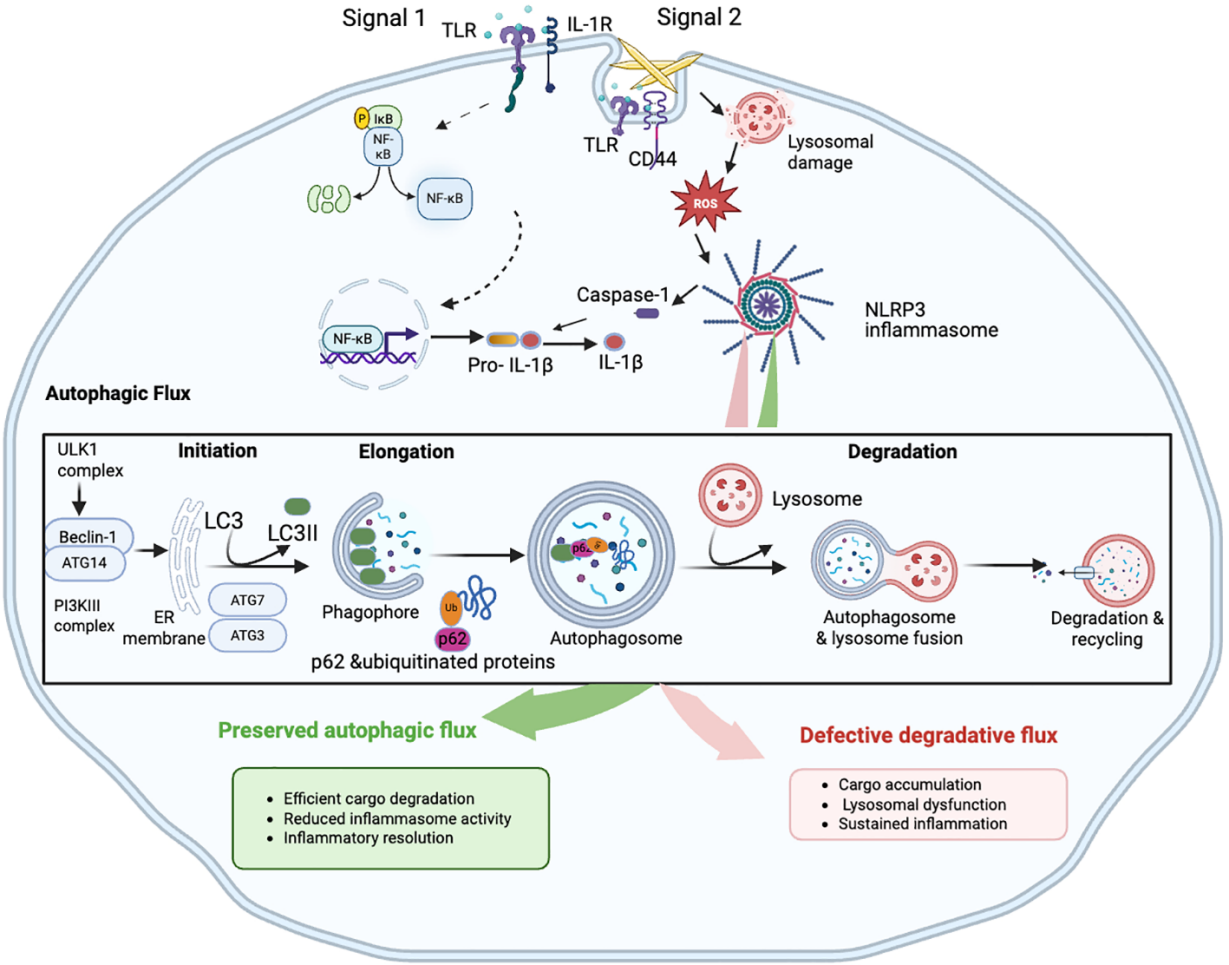
Proposed model of autophagic flux and inflammasome signaling in gouty arthritis (GA). Priming of synovial immune cells, chondrocytes, or osteoblasts by damage-associated molecular patterns (DAMPs), microbial ligands, or IL-1β through pattern-recognition receptors (PRRs) or the IL-1 receptor (Signal 1) may promote NF-κB activation, pro-IL-1β expression, and NLRP3 inflammasome activation. Subsequent exposure to monosodium urate (MSU) crystals (Signal 2) is associated with the generation of reactive oxygen species (ROS) and activation of the NLRP3 inflammasome, leading to caspase-1 activation and IL-1β maturation. Autophagy may be activated in parallel and contributes to intracellular cargo turnover via lysosomal degradation. Preserved autophagic flux is associated with efficient cargo degradation and reduced inflammasome activity, whereas defective degradative flux is characterized by cargo accumulation, lysosomal dysfunction, and persistent inflammatory signaling. The biological consequences of autophagy may vary according to cell type, disease stage, and the integrity of autophagic flux. (Created with https://BioRender.com/).

Although pharmacological modulation of autophagy has demonstrated promising anti-inflammatory effects in experimental models, translation into clinical practice remains challenging because of disease heterogeneity, cell-specific responses, and the pleiotropic actions of many currently available compounds. Future studies should prioritize rigorous assessment of autophagic flux, validation in clinically relevant chronic disease models and human tissues, and identification of selective therapeutic approaches capable of restoring autophagic and lysosomal homeostasis. An improved understanding of the cell- and stage-specific regulation of autophagy may facilitate the development of more precise therapeutic strategies for gout. A schematic overview of the proposed relationship between autophagic flux integrity, NLRP3 inflammasome activation, and inflammatory outcomes in GA is presented in **Figure 1**.

## References

[1] Bitik B Öztürk MA . An old disease with new insights: Update on diagnosis and treatment of gout. Eur J Rheumatol. (2014) 1:72. doi: 10.5152/eurjrheum.2014.021 27708879 PMC5042282

[2] Busso N So A . Gout. Mechanisms of inflammation in gout. Arthritis Res Ther. (2010) 12:206. doi: 10.1186/ar2952 20441605 PMC2888190

[3] Pascual E Addadi L Andrés M Sivera F . Mechanisms of crystal formation in gout—a structural approach. Nat Rev Rheumatol. (2015) 11:725–30. doi: 10.1038/nrrheum.2015.125 26369610

[4] Roddy E Choi H . Epidemiology of gout. Rheumatic Dis Clinics North America. (2014) 40:155. doi: 10.1186/ar3199 24703341 PMC4119792

[5] Martin WJ Walton M Harper J . Resident macrophages initiating and driving inflammation in a monosodium urate monohydrate crystal–induced murine peritoneal model of acute gout. Arthritis Rheumatism. (2009) 60:281–9. doi: 10.1002/art.24185 19116939

[6] So AK Martinon F . Inflammation in gout: mechanisms and therapeutic targets. Nat Rev Rheumatol. (2017) 13:639–47. doi: 10.1038/nrrheum.2017.155 28959043

[7] Dalbeth N Choi HK Joosten LAB Khanna PP Matsuo H Perez-Ruiz F . Gout (primer). Nat Rev Dis Primers. (2019) 5(1):1–21. doi: 10.1093/med/9780199642489.003.0141 31558729

[8] Narang RK Dalbeth N . Management of complex gout in clinical practice: update on therapeutic approaches. Best Pract Res Clin Rheumatol. (2018) 32:813–34. doi: 10.1016/j.berh.2019.03.010 31427057

[9] Klionsky DJ . Autophagy: from phenomenology to molecular understanding in less than a decade. Nat Rev Mol Cell Biol. (2007) 8:931–7. doi: 10.1038/nrm2245 17712358

[10] Mizushima N Komatsu M . Autophagy: renovation of cells and tissues. Cell. (2011) 147:728–41. doi: 10.1016/j.cell.2011.10.026 22078875

[11] Hsieh CY Li LH Lam Y Fang Z Gan CH Rao YK . Synthetic 4-hydroxy auxarconjugatin B, a novel autophagy inducer, attenuates gouty inflammation by inhibiting the NLRP3 inflammasome. Cells. (2020) 9:279. doi: 10.3390/cells9020279 31979265 PMC7072356

[12] Huang S Wang Y Lin S Guan W Liang H Shen J . Neutrophil autophagy induced by monosodium urate crystals facilitates neutrophil extracellular traps formation and inflammation remission in gouty arthritis. Front Endocrinol. (2023) 14:1071630. doi: 10.3389/fendo.2023.1071630 37810893 PMC10557066

[13] Nie J Qiu H . DUSP1 mitigates MSU-induced immune response in gouty arthritis reinforcing autophagy. Front Bioscience-Landmark. (2024) 29:222. doi: 10.31083/j.fbl2906222 38940057

[14] Yang QB He YL Zhong XW Xie WG Zhou JG . Resveratrol ameliorates gouty inflammation via upregulation of sirtuin 1 to promote autophagy in gout patients: Q.-B. Yang. Inflammopharmacology. (2019) 27:47–56. doi: 10.1007/s10787-018-00555-4 30600470

[15] Allaeys I Marceau F Poubelle PE . NLRP3 promotes autophagy of urate crystals phagocytized by human osteoblasts. Arthritis Res Ther. (2013) 15:R176. doi: 10.1186/ar4365 24456929 PMC4061723

[16] Chen YH Chen WY Yu CL Tsai CY Hsieh SC . Gouty arthritis involves impairment of autophagic degradation via cathepsin D inactivation-mediated lysosomal dysfunction that promotes apoptosis in macrophages. Biochim Biophys Acta (BBA)-Mol Basis Dis. (2023) 1869:166703. doi: 10.1016/j.bbadis.2023.166703 37001704

[17] Choe JY Jung HY Park KY Kim SK . Enhanced p62 expression through impaired proteasomal degradation is involved in caspase-1 activation in monosodium urate crystal-induced interleukin-1β expression. Rheumatology. (2014) 53:1043–53. doi: 10.1093/rheumatology/ket474 24587486

[18] Fu W Ge M Li J . Phospholipase A2 regulates autophagy in gouty arthritis: proteomic and metabolomic studies. J Transl Med. (2023) 21:261. doi: 10.1186/s12967-023-04114-6 37069596 PMC10108447

[19] Hwang HS Yang CM Park SJ Kim HA . Monosodium urate crystal-induced chondrocyte death via autophagic process. Int J Mol Sci. (2015) 16:29265–77. doi: 10.3390/ijms161226164 26670233 PMC4691108

[20] Kim S-K Choe J-Y Kim H . SAT0385 monosodium urate crystal-induced interleukin-1Beta expression is related to p62 level in autophagy process. Ann Rheumatic Dis. (2013) 72:A714. doi: 10.1136/annrheumdis-2013-eular.2110

[21] Xiao L Lin S Xu W Sun E . Downregulation of Sox8 mediates monosodium urate crystal-induced autophagic impairment of cartilage in gout arthritis. Cell Death Discov. (2023) 9:95. doi: 10.1038/s41420-023-01388-z 36918540 PMC10015026

[22] Guo J Lei T Jiang Y Wang P Zhang Z Yu X . Circ_0058051 targeted miR‐129‐5P regulates autophagy‐Related gene ATG7 to promote the inflammation of gout. Mediators Inflammation. (2025) 2025:6645479. doi: 10.1155/mi/6645479 PMC12602026 41221017

[23] Zhang XJ Chen S Huang KX Le WD . Why should autophagic flux be assessed? Acta Pharmacol Sin. (2013) 34:595–9. doi: 10.1038/aps.2012.184 23474710 PMC4002868

[24] Parzych KR Klionsky DJ . An overview of autophagy: morphology, mechanism, and regulation. Antioxid Redox Signaling. (2014) 20:460–73. doi: 10.1089/ars.2013.5371 23725295 PMC3894687

[25] Alers S Löffler AS Wesselborg S Stork B . Role of AMPK-mTOR-Ulk1/2 in the regulation of autophagy: cross talk, shortcuts, and feedbacks. Mol Cell Biol. (2012) 32:2–11. doi: 10.1128/mcb.06159-11 22025673 PMC3255710

[26] Coutts AS La Thangue NB . Regulation of actin nucleation and autophagosome formation. Cell Mol Life Sci. (2016) 73:3249–63. doi: 10.1007/s00018-016-2224-z 27147468 PMC4967107

[27] Kotani T Nakatogawa H . Core principles of autophagy initiation mechanisms. Nat Struct Mol Biol. (2026) 33:408–419. doi: 10.1038/s41594-026-01752-4 41721018

[28] Geng J Klionsky DJ . The Atg8 and Atg12 ubiquitin‐like conjugation systems in macroautophagy. EMBO Rep. (2008) 9:859–64. doi: 10.1038/embor.2008.163 18704115 PMC2529362

[29] Ohsumi Y . Molecular dissection of autophagy: two ubiquitin-like systems. Nat Rev Mol Cell Biol. (2001) 2:211–6. doi: 10.1038/35056522 11265251

[30] Zheng YT Shahnazari S Brech A Lamark T Johansen T Brumell JH . The adaptor protein p62/SQSTM1 targets invading bacteria to the autophagy pathway. J Immunol. (2009) 183:5909–16. doi: 10.4049/jimmunol.0900441 19812211

[31] Crişan TO Cleophas MCP Novakovic B Erler K van de Veerdonk FL Stunnenberg HG . Uric acid priming in human monocytes is driven by the AKT–PRAS40 autophagy pathway. Proc Natl Acad Sci. (2017) 114:5485–90. doi: 10.1073/pnas.1620910114 PMC5448210 28484006

[32] Mitroulis I Kambas K Chrysanthopoulou A Skendros P Apostolidou E Kourtzelis I . Neutrophil extracellular trap formation is associated with IL-1β and autophagy-related signaling in gout. PLoS One. (2011) 6:e29318. doi: 10.1371/journal.pone.0029318 22195044 PMC3241704

[33] Chittaranjan S Bortnik S Gorski SM . Monitoring autophagic flux by using lysosomal inhibitors and western blotting of endogenous MAP1LC3B. Cold Spring Harbor Protoc. (2015) 2015:pdb. prot086256. doi: 10.1101/pdb.prot086256 26240408

[34] Giménez-Xavier P Francisco R Platini F Pérez R Ambrosio S . LC3-I conversion to LC3-II does not necessarily result in complete autophagy. Int J Mol Med. (2008) 22:781–5. Available online at: https://pubmed.ncbi.nlm.nih.gov/19020776/ 19020776

[35] Mizushima N Yoshimori T . How to interpret LC3 immunoblotting. Autophagy. (2007) 3:542–5. doi: 10.4161/auto.4600 17611390

[36] Kim JY Ozato K . The sequestosome 1/p62 attenuates cytokine gene expression in activated macrophages by inhibiting IFN regulatory factor 8 and TNF receptor-associated factor 6/NF-κB activity. J Immunol. (2009) 182:2131–40. doi: 10.4049/jimmunol.0802755 19201866 PMC4151355

[37] Kim MJ Min Y Kwon J Son J Im JS Shin J . p62 negatively regulates TLR4 signaling via functional regulation of the TRAF6-ECSIT complex. Immune Network. (2019) 19(3):1–14. doi: 10.4110/in.2019.19.e16 31281713 PMC6597446

[38] Wong W . Limiting inflammation with p62. Sci Signaling. (2016) 9:ec52–2. doi: 10.1126/scisignal.aaf6257

[39] Barth S Glick D Macleod KF . Autophagy: assays and artifacts. J Pathol. (2010) 221:117–24. doi: 10.1002/path.2694 20225337 PMC2989884

[40] Oh SH Choi YB Kim JH Weihl CC Ju JS . Quantification of autophagy flux using LC3 ELISA. Anal Biochem. (2017) 530:57–67. doi: 10.1016/j.ab.2017.05.003 28477964

[41] Hahn J Knopf J Maueröder C Kienhöfer D Leppkes M Herrmann M . Neutrophils and neutrophil extracellular traps orchestrate initiation and resolution of inflammation. Clin Exp Rheumatol. (2016) 34:6–8. Available online at: https://pubmed.ncbi.nlm.nih.gov/27586795/ 27586795

[42] Lee KH Kronbichler A Park DD Park Y Moon H Kim H . Neutrophil extracellular traps (NETs) in autoimmune diseases: a comprehensive review. Autoimmun Rev. (2017) 16:1160–73. doi: 10.1016/j.autrev.2017.09.012 28899799

[43] Li C Wu C Li F Xu W Zhang X Huang Y . Targeting neutrophil extracellular traps in gouty arthritis: insights into pathogenesis and therapeutic potential. J Inflammation Res. (2024) 17:1735–63. doi: 10.2147/jir.s460333 38523684 PMC10960513

[44] Abraham S Clark A . Dual-specificity phosphatase 1: a critical regulator of innate immune responses. Biochem Soc Trans. (2006) 34:1018–23. doi: 10.1042/bst0341018 17073741

[45] Baur JA . Resveratrol, sirtuins, and the promise of a DR mimetic. Mech Ageing Dev. (2010) 131:261–9. doi: 10.1016/j.mad.2010.02.007 20219519 PMC2862768

[46] Claude-Taupin A Isnard P Bagattin A Kuperwasser N Roccio F Ruscica B . The AMPK-Sirtuin 1-YAP axis is regulated by fluid flow intensity and controls autophagy flux in kidney epithelial cells. Nat Commun. (2023) 14:8056. doi: 10.1038/s41467-023-43775-1 38052799 PMC10698145

[47] Son Y Han M Wu X Roh YS . SIRT1-mediated redox and senescence regulation in cancer: mechanisms and therapeutic implications. Antioxidants. (2025) 14:1076. doi: 10.3390/antiox14091076 41008982 PMC12466425

[48] Wang Y Li Y Ding H Li D Shen W Zhang X . The current state of research on sirtuin-mediated autophagy in cardiovascular diseases. J Cardiovasc Dev Dis. (2023) 10:382. doi: 10.3390/jcdd10090382 37754811 PMC10531599

[49] Xu J Jackson CW Khoury N Escobar I Perez-Pinzon MA . Brain SIRT1 mediates metabolic homeostasis and neuroprotection. Front Endocrinol. (2018) 9:702. doi: 10.3389/fendo.2018.00702 30532738 PMC6265504

[50] Yang Y Liu Y Wang Y Chao Y Zhang J Jia Y . Regulation of SIRT1 and its roles in inflammation. Front Immunol. (2022) 13:831168. doi: 10.3389/fimmu.2022.831168 35359990 PMC8962665

[51] Wiza C Nascimento EB Ouwens DM . Role of PRAS40 in Akt and mTOR signaling in health and disease. Am J Physiol Endocrinol Metab. (2012) 302:E1453–60. doi: 10.1152/ajpendo.00660.2011 22354785

[52] Tsang KY Cheah KS . The extended chondrocyte lineage: implications for skeletal homeostasis and disorders. Curr Opin Cell Biol. (2019) 61:132–40. doi: 10.1016/j.ceb.2019.07.011 31541943

[53] Yildirim N Amanzhanova A Kulzhanova G Mukasheva F Erisken C . Osteochondral interface: regenerative engineering and challenges. ACS Biomater Sci Eng. (2023) 9:1205–23. doi: 10.1021/acsbiomaterials.2c01321 36752057

[54] Biasizzo M Kopitar-Jerala N . Interplay between NLRP3 inflammasome and autophagy. Front Immunol. (2020) 11:591803. doi: 10.3389/fimmu.2020.591803 33163006 PMC7583715

[55] Paik S Kim JK Silwal P Sasakawa C Jo EK . An update on the regulatory mechanisms of NLRP3 inflammasome activation. Cell Mol Immunol. (2021) 18:1141–60. doi: 10.1038/s41423-021-00670-3 33850310 PMC8093260

[56] Leidal AM Debnath J . Unraveling the mechanisms that specify molecules for secretion in extracellular vesicles. Methods. (2020) 177:15–26. doi: 10.1016/j.ymeth.2020.01.008 31978536 PMC7198338

[57] Longo A Manganelli V Misasi R Riitano G Caglar TR Fasciolo E . Extracellular vesicles in the crosstalk of autophagy and apoptosis: A role for lipid rafts. Cells. (2025) 14:749. doi: 10.3390/cells14100749 40422252 PMC12109985

[58] Ponpuak M Mandell MA Kimura T Chauhan S Cleyrat C Deretic V . Secretory autophagy. Curr Opin Cell Biol. (2015) 35:106–16. doi: 10.1016/j.ceb.2015.04.016 25988755 PMC4529791

[59] Manganelli V Dini L Tacconi S Dinarelli S Capozzi A Riitano G . Autophagy promotes enrichment of raft components within extracellular vesicles secreted by human 2FTGH cells. Int J Mol Sci. (2024) 25:6175. doi: 10.3390/ijms25116175 38892363 PMC11172899

[60] Liu Z Chu A Bai Z Yang C . Daphnetin alleviates inflammation and promotes autophagy via the AMPK/mTOR pathway in gouty arthritis. J Cell Commun Signaling. (2025) 19:e70011. doi: 10.1002/ccs3.70011 40304008 PMC12037417

[61] Liu Z Chu A Bai Z Yang C . Nobiletin ameliorates monosodium urate-induced gouty arthritis in mice by enhancing AMPK/mTOR-mediated autophagy to inhibit NF-κB/NLRP3 inflammasome activation. Immunol Lett. (2025) 274:106982. doi: 10.1016/j.imlet.2025.106982 39965668

[62] Lou D Zhang X Jiang C Zhang F Xu C Fang S . 3β, 23‐dihydroxy‐12‐ene‐28‐ursolic acid isolated from Cyclocarya paliurus alleviates NLRP3 inflammasome‐mediated gout via PI3K‐AKT‐mTOR‐dependent autophagy. Evidence‐Based Complementary Altern Med. (2022) 2022:5541232. doi: 10.1155/2022/5541232 PMC8763513 35047046

[63] Han J Shi G Li W Wang S Bai J Sun X . Zisheng Shenqi decoction ameliorates monosodium urate‐mediated gouty arthritis in rats via promotion of autophagy through the AMPK/mTOR signaling pathway. Evidence‐Based Complementary Altern Med. (2021) 2021:6918026. doi: 10.1155/2021/6918026 33505502 PMC7806400

[64] Liu P Xu Y Ye J Tan J Hou J Wang Y . Qingre Huazhuo Jiangsuan Decoction promotes autophagy by inhibiting PI3K/AKT/mTOR signaling pathway to relieve acute gouty arthritis. J Ethnopharmacol. (2023) 302:115875. doi: 10.1016/j.jep.2022.115875 36328206

[65] Du Y Zhang Y Jiang Z Xu L Ru J Wei S . Triptolide alleviates acute gouty arthritis caused by monosodium urate crystals by modulating macrophage polarization and neutrophil activity. Immunol Lett. (2024) 269:106907. doi: 10.1016/j.imlet.2024.106907 39122094

[66] Yuan W Liu T Wang YY He S Zhang F Wang XB . Autophagy induced by PP121 alleviates MSU crystal-induced acute gouty arthritis via inhibition of the NLRP3 inflammasome. Int Immunopharmacol. (2023) 123:110756. doi: 10.1016/j.intimp.2023.110756 37573689

[67] Piao MH Wang H Jiang YJ Wu YL Nan JX Lian LH . Taxifolin blocks monosodium urate crystal-induced gouty inflammation by regulating phagocytosis and autophagy. Inflammopharmacology. (2022) 30:1335–49. doi: 10.1007/s10787-022-01014-x 35708797

[68] Xu L Liu X Zhang Y Jia T Li L Du Y . Tanshinone IIA improves acute gouty arthritis in rats through regulating neutrophil activation and the NLRP3 inflammasome. Dis Markers. (2022) 2022:5851412. doi: 10.1155/2022/5851412 36578443 PMC9792249

